# Supplementary material for: Deletion of wfs1 Impairs Oligodendrocyte Precursor Cells Dorsal Distribution and Myelination Through the wfs1-hmgcs1 Axis in Zebrafish
Source: Biology (Basel). 2026 Aug 16;15(16):1402. doi: 10.3390/biology15161402 (PMC13510151; doi:10.3390/biology15161402)
Supplement: Supplementary file 1 [file biology-15-01402-s001.zip › File S1-Transcriptome sequencing data and differentially expressed genes (DEGs) in wfs1-- zebrafish at 5 dpf/1.GO/wfs1KOvsWT/GSEA/WT/CELL_DIVISION_SITE/CELL_DIVISION_SITE(GO_0032153).html]

Details for gene set CELL\_DIVISION\_SITE(GO:0032153)[GSEA]


|  || Dataset | deg.sample |
| Phenotype | sample.cls |
| Upregulated in class | WT |
| GeneSet | CELL\_DIVISION\_SITE(GO:0032153) |
| Enrichment Score (ES) | -0.48505467 |
| Normalized Enrichment Score (NES) | -2.3037324 |
| Nominal p-value | 0.0 |
| FDR q-value | 0.060728014 |
| FWER p-Value | 0.012 |
Table: GSEA Results Summary

  

Fig 1: Enrichment plot: CELL\_DIVISION\_SITE(GO:0032153)
  
Profile of the Running ES Score & Positions of GeneSet Members on the Rank Ordered List

  

| PROBE | DESCRIPTION   (from dataset) | GENE SYMBOL | GENE\_TITLE | RANK IN GENE LIST | RANK METRIC SCORE | RUNNING ES | CORE ENRICHMENT || 1 | 567134 | zgc:153916 |  |  | 4472 | 0.749 | -0.1107 | No |
| 2 | 323182 | pkn3 |  |  | 6375 | 0.549 | -0.1486 | No |
| 3 | 556486 | sept4a |  |  | 6511 | 0.536 | -0.1316 | No |
| 4 | 573631 | rab11fip3 |  |  | 6523 | 0.535 | -0.1107 | No |
| 5 | 797422 | stambpa |  |  | 6935 | 0.497 | -0.1038 | No |
| 6 | 80959 | pard6gb |  |  | 7495 | 0.454 | -0.1033 | No |
| 7 | 550339 | wdr73 |  |  | 7586 | 0.449 | -0.0883 | No |
| 8 | 571965 | pkn2 |  |  | 8676 | 0.389 | -0.1070 | No |
| 9 | 394136 | sept7a |  |  | 9042 | 0.365 | -0.1040 | No |
| 10 | 445215 | mastl |  |  | 9083 | 0.361 | -0.0909 | No |
| 11 | 334375 | buc |  |  | 9611 | 0.326 | -0.0945 | No |
| 12 | 563517 | tubgcp3 |  |  | 9783 | 0.317 | -0.0873 | No |
| 13 | 571702 | sept8b |  |  | 9890 | 0.310 | -0.0783 | No |
| 14 | 436685 | zfyve19 |  |  | 10603 | 0.266 | -0.0901 | No |
| 15 | 553191 | tubgcp5 |  |  | 11649 | 0.208 | -0.1146 | No |
| 16 | 447899 | rtkn2b |  |  | 11830 | 0.199 | -0.1123 | No |
| 17 | 566722 | fsd1 |  |  | 12126 | 0.183 | -0.1143 | No |
| 18 | 562517 | anln |  |  | 12145 | 0.182 | -0.1077 | No |
| 19 | 445325 | sept5b |  |  | 12645 | 0.157 | -0.1171 | No |
| 20 | 30627 | kif23 |  |  | 13079 | 0.136 | -0.1253 | No |
| 21 | 323457 | sept2 |  |  | 14860 | 0.053 | -0.1791 | No |
| 22 | 335925 | sept5a |  |  | 15509 | 0.022 | -0.1986 | No |
| 23 | 386975 | tubgcp2 |  |  | 16854 | -0.028 | -0.2397 | No |
| 24 | 557155 | tubgcp4 |  |  | 16954 | -0.032 | -0.2415 | No |
| 25 | 564545 | si:dkey-40c11.1 |  |  | 17126 | -0.037 | -0.2454 | No |
| 26 | 550129 | sept10 |  |  | 18262 | -0.088 | -0.2775 | No |
| 27 | 569040 | plcd3a |  |  | 18746 | -0.111 | -0.2883 | No |
| 28 | 321590 | sept8a |  |  | 19001 | -0.121 | -0.2914 | No |
| 29 | 554123 | sept3 |  |  | 19212 | -0.131 | -0.2928 | No |
| 30 | 641572 | stambpb |  |  | 19281 | -0.135 | -0.2896 | No |
| 31 | 336281 | vamp2 |  |  | 19711 | -0.153 | -0.2970 | No |
| 32 | 100000597 | sept7b |  |  | 20105 | -0.171 | -0.3026 | No |
| 33 | 767727 | zgc:154045 |  |  | 20322 | -0.180 | -0.3022 | No |
| 34 | 393431 | zgc:63587 |  |  | 20871 | -0.208 | -0.3111 | No |
| 35 | 436806 | rab11fip4a |  |  | 20998 | -0.215 | -0.3065 | No |
| 36 | 337243 | sept9a |  |  | 21278 | -0.229 | -0.3062 | No |
| 37 | 564600 | sept12 |  |  | 22004 | -0.267 | -0.3183 | No |
| 38 | 553800 | cep55l |  |  | 22351 | -0.287 | -0.3178 | No |
| 39 | 559353 | si:dkey-30c15.10 |  |  | 22400 | -0.290 | -0.3078 | No |
| 40 | 322694 | sept6 |  |  | 23764 | -0.378 | -0.3356 | No |
| 41 | 100006041 | rhoab |  |  | 24773 | -0.436 | -0.3499 | No |
| 42 | 558037 | zgc:162239 |  |  | 25031 | -0.449 | -0.3402 | No |
| 43 | 100007341 | si:ch1073-365p7.2 |  |  | 29041 | -0.929 | -0.4292 | Yes |
| 44 | 100151129 | rab11fip4b |  |  | 30821 | -1.778 | -0.4144 | Yes |
| 45 | 557962 | spire1a |  |  | 30915 | -1.880 | -0.3426 | Yes |
| 46 | 792259 | sept15 |  |  | 31355 | -2.543 | -0.2554 | Yes |
| 47 | 557239 | antxr2a |  |  | 31582 | -3.209 | -0.1350 | Yes |
| 48 | 541494 | sept9b |  |  | 31689 | -3.641 | 0.0063 | Yes |
Table: GSEA details
[plain text format]

  

Fig 2: CELL\_DIVISION\_SITE(GO:0032153)
  
Blue-Pink O' Gram in the Space of the Analyzed GeneSet

  

Fig 3: CELL\_DIVISION\_SITE(GO:0032153): Random ES distribution
  
Gene set null distribution of ES for
 **CELL\_DIVISION\_SITE(GO:0032153)**
